# Understanding the Intention to Use Telehealth Services in Underserved Hispanic Border Communities: Cross-Sectional Study

**DOI:** 10.2196/21012

**Published:** 2020-09-03

**Authors:** Suad Ghaddar, Kristina P Vatcheva, Samantha G Alvarado, Laryssa Mykyta

**Affiliations:** 1 Department of Health and Biomedical Sciences University of Texas Rio Grande Valley Edinburg, TX United States; 2 School of Mathematical and Statistical Sciences University of Texas Rio Grande Valley Brownsville, TX United States; 3 School of Medicine University of Texas Rio Grande Valley Edinburg, TX United States; 4 Social, Economic and Housing Statistics Division Health and Disability Statistics Branch US Census Bureau Washington, DC United States

**Keywords:** telehealth, eHealth literacy, health information technologies

## Abstract

**Background:**

Despite the United States having one of the leading health care systems in the world, underserved minority communities face significant access challenges. These communities can benefit from telehealth innovations that promise to improve health care access and, consequently, health outcomes. However, little is known about the attitudes toward telehealth in these communities, an essential first step toward effective adoption and use.

**Objective:**

The purpose of this study is to assess the factors that shape behavioral intention to use telehealth services in underserved Hispanic communities along the Texas-Mexico border and examine the role of electronic health (eHealth) literacy in telehealth use intention.

**Methods:**

We used cross-sectional design to collect data at a community health event along the Texas-Mexico border. The area is characterized by high poverty rates, low educational attainment, and health care access challenges. Trained bilingual students conducted 322 in-person interviews over a 1-week period. The survey instrument assessed sociodemographic information and telehealth-related variables. Attitudes toward telehealth were measured by asking participants to indicate their level of agreement with 9 statements reflecting different aspects of telehealth use. For eHealth literacy, we used the eHealth Literacy Scale (eHEALS), an 8-item scale designed to measure consumer confidence in finding, evaluating, and acting upon eHealth information. To assess the intention to use telehealth, we asked participants about the likelihood that they would use telehealth services if offered by a health care provider. We analyzed data using univariate, multivariate, and mediation statistical models.

**Results:**

Participants were primarily Hispanic (310/319, 97.2%) and female (261/322, 81.1%), with an average age of 43 years. Almost three-quarters (219/298) reported annual household incomes below $20,000. Health-wise, 42.2% (136/322) self-rated their health as fair or poor, and 79.7% (255/320) were uninsured. The overwhelming majority (289/319, 90.6%) had never heard of telehealth. Once we defined the term, participants exhibited positive attitudes toward telehealth, and 78.9% (254/322) reported being somewhat likely or very likely to use telehealth services if offered by a health care provider. Based on multivariate proportional odds regression analysis, a 1-point increase in telehealth attitudes reduced the odds of lower versus higher response in the intention to use telehealth services by 23% (OR 0.77, 95% CI 0.73-0.81). Mediation analysis revealed that telehealth attitudes fully mediated the association between eHealth literacy and intention to use telehealth services. For a 1-point increase in eHEALS, the odds of lower telehealth use decreased by a factor of 0.95 (5%; OR 0.95, 95% CI 0.93-0.98; *P*<.001) via the increase in the score of telehealth attitudes.

**Conclusions:**

Telehealth promises to address many of the access challenges facing ethnic and racial minorities, rural communities, and low-income populations. Findings underscore the importance of raising awareness of telehealth and promoting eHealth literacy as a key step in fostering positive attitudes toward telehealth and furthering interest in its use.

## Introduction

The United States has one of the leading health care systems in the world, offering highly specialized and technologically advanced medical care. At the same time, the US health care system faces many challenges, especially when serving vulnerable communities (eg, low socioeconomic status groups, minority populations, and uninsured people). Primary among these challenges is access to care, including lack of health insurance coverage [1], shortages of primary and specialty care providers [2], transportation difficulties [3], and language barriers [4,5], among others. With recent advances in technology, telehealth promises to address many of these access challenges.

Telehealth is commonly defined as “the use of electronic information and telecommunications technologies to support and promote long-distance clinical health care, patient and professional health-related education, public health, and health administration” [6]. This definition encompasses a broad scope of remote health care services (eg, telemedicine, telemonitoring, mobile health [mHealth] apps, patient portals). For the purposes of this study, telehealth as presented to participants and supported by the statements assessing their attitudes is more representative of telemedicine rather than other health information technologies (HIT). We opted to use the term telehealth in the survey because of its broader scope and higher likelihood of public recognition.

Several models, such as the technology acceptance model [7] and the unified theory of acceptance and use of technology [8], have been developed to depict consumer interest and willingness to use technology. With the expansion of technology into the health care sector, these models, with various modifications, have been applied to the adoption of HIT such as mHealth apps, patient portals, telemonitoring, and telemedicine [9]. Only recently have efforts been directed at developing technology use models specific to the health context [10,11]. In addition to the common key concepts across previous models (attitudes, behavioral intention, and behavior), HIT-specific models expand the focus from the technology’s features to incorporate end-user characteristics (eg, health status, internet self-efficacy) and the realm of social influence. The role of electronic health (eHealth) literacy, commonly defined as “consumers’ combined knowledge, comfort, and perceived skills at finding, evaluating, and applying electronic health information to health problems” [12], has rarely been integrated in these models [11]. Yet evidence has been mounting in support of its role in the adoption of various HIT apps such as patient portals [13,14] and mHealth [15,16]. In line with conceptual models depicting end-user characteristics influencing behavioral intention to use HIT through the mediating effects of perceived ease of use, perceived usefulness, and attitudes [10], we hypothesize that eHealth literacy will exhibit a similar indirect effect on the behavioral intention to use telehealth.

Most telehealth research in the United States has initially focused on telehealth adoption from the perspective of health care providers [17-21] and health systems [20,22] or on policies [23] and reimbursement models that facilitate its adoption [24]. The consumer/patient perspective has just recently been more extensively considered. However, most patient research on telehealth has been conducted at the international level [21,25,26] or has focused on white, non-Hispanic populations within the United States [27]. The perspective and characteristics of individuals from vulnerable US minority communities has not received much attention, although there are a few notable exceptions focused primarily on African Americans [28,29]. Given that Hispanics are the largest minority group in the United States [30], it is important to understand the factors that influence their acceptance of telehealth, especially given that acceptance is a predictor of adoption [31]. This entails examining several dimensions, primary among which are end-user characteristics and attitudes toward health technology. Thus, the purpose of this study is to assess the factors that shape behavioral intentions to use telehealth services in vulnerable, marginalized Hispanic communities along the Texas-Mexico border and examine the role of eHealth literacy in telehealth intention use.

## Methods

### Study Setting

We collected data from participants at Operation Lone Star (OLS), a joint military and civilian public health emergency preparedness exercise that takes place annually along the Texas-Mexico border. OLS is a cooperative effort between the Texas Department of State Health Services, Cameron County Public Health Department, Hidalgo County Health and Human Services, City of Laredo Health Department, the Texas State Guard, and various community volunteer organizations. The event brings free health care services to area residents. These include child immunizations, sports physicals, hearing screenings, vision screenings for prescription glasses, diabetes and blood pressure screenings, and dental services, among others.

In 2018, OLS events took place during the week of July 23-27 at 6 locations across 4 border counties (Cameron, Hidalgo, Starr, and Webb). For this study, we collected data at one of the two Hidalgo County sites, which provided services to 2294 children and adult county residents over the course of OLS week. Hidalgo County is the largest county along the Texas-Mexico border; it is home to almost 850,000 people, the overwhelming majority of whom are of Hispanic or Latino origin (92%) [30]. The county is characterized by high poverty rates (almost a third of the population lives below the federal poverty level) and low educational attainment (36% of individuals aged 25 years and over do not have a high school degree) [30]. Lack of health care coverage is a major access challenge with 43% of individuals aged 18 to 64 years being uninsured in 2018 [32].

### Recruitment and Data Collection

Data were collected in person by students participating in a special course-based undergraduate research experience, two graduate research assistants, and the first author. All data collection team members completed training in the ethical conduct of research, survey administration, and interviewing techniques, as well as additional requirements for participation at OLS. Most team members were bilingual (English and Spanish).

We employed a convenience sampling design to recruit participants. The data collection team approached event attendees waiting to receive health services at various stations, provided them with information about the study and invited them to participate. The 15- to 20-minute interviews were conducted in either English or Spanish, based on the participant’s preferred language. After completing the anonymous interview, participants were provided with a drawstring bag, a bottle of water, and a chance to enter in a raffle for one of sixty $50 gift cards from a local grocery store.

All study procedures were approved by the institutional review board at the University of Texas Rio Grande Valley (UTRGV).

### Survey Instrument

The survey instrument included questions assessing sociodemographic information, health status, eHealth literacy, and telehealth-related variables measuring attitudes and behavioral intentions to use telehealth. For sociodemographic characteristics and health status variables, we used questions from existing national surveys (eg, US Census Bureau, Centers for Disease Control and Prevention [CDC]) for which existing Spanish translation was available. For the remaining variables, where no Spanish translation was available, a bilingual (English and Spanish) graduate student translated the survey. The survey was then piloted by bilingual and native Spanish-speaking interviewers who assessed participant understanding of both the English and Spanish versions. Minor modifications were made to reflect the area’s culture and local linguistic Spanish use.

We provided participants with the following definition before asking about the two main telehealth-related variables (outcome measure: behavioral intention to use telehealth; predictor measure: attitudes toward telehealth): Telehealth uses technology to access and manage health care outside of doctors’ offices or clinics. Some examples are receiving care from your health care provider by video, remote monitoring of blood pressure or heart rate, or checking your laboratory results online.

### Outcome Measure: Behavioral Intention to Use Telehealth Services

We assessed the behavioral intention to use telehealth by the question, “How likely are you to use telehealth services if they were offered by your provider?” Response options included: very likely, somewhat likely, not very likely, and would not use telehealth services.

### Predictor Measures

#### Attitudes Toward Telehealth

We assessed attitudes toward telehealth by asking respondents to rate their level of agreement (5-point Likert scale: 1=strongly disagree to 5=strongly agree) with 9 statements reflecting different aspects of telehealth use such as perceived ease of use, perceived usefulness, and perceived cost effectiveness. The statements were adopted with minor modifications from a study on patient telemedicine readiness in a Louisiana oncology practice [28]; the instrument was developed based on the technology acceptance model [7,33] and the fit between individuals, task, and technology framework [34]. The summated scale (range 9 to 45 with higher scores reflecting more positive attitudes) demonstrated good internal consistency (Cronbach alpha of .794); the internal consistency was lower for the Spanish surveys (Cronbach alpha of .757) as compared with the English surveys (Cronbach alpha of .839) but still above the acceptable .70 threshold value [35].

#### Telehealth Readiness

We assessed telehealth readiness with 3 questions related to the methods participants used to (1) make an appointment with their health care provider, (2) communicate with their health care provider, and (3) keep track of their personal health information. A participant was considered telehealth-ready if they had communicated with their health care provider to make an appointment or discussed their test results via a website and/or email or kept track of their personal health information using an online system.

### eHealth Literacy

We used the 8-item eHealth Literacy Scale (eHEALS) to assess eHealth literacy [12]. For each item, respondents indicated their level of agreement on a 5-point Likert scale (1=strongly disagree to 5=strongly agree). Higher scores on the summation of responses (range 8 to 40) reflect higher levels of eHealth literacy. The reliability and validity of eHEALS has been previously established in both English and Spanish [12,36]. Cronbach alpha, assessing the internal consistency of eHEALS, was .916 for our sample (.904 and .924 for the English- and Spanish-speaking subsamples, respectively).

### Sociodemographic Variables

We assessed several sociodemographic variables, including age, sex, employment status, marital status, educational attainment, country of birth, ethnicity, annual household income, and health care coverage.

### Internet Knowledge and Skills

We used 2 questions to examine participant internet knowledge and skills. The first question (dichotomized yes/no) asked participants whether they have “ever gone online to access the internet or to send and receive emails.” The second question asked respondents to self-rate their internet skills. Responses were dichotomized as yes=fairly skilled, very skilled, or expert and no=not at all skilled or not very skilled.

### Health Status

To assess the health of participants, we used 2 measures. The first measure examined general health status through the CDC’s validated self-rated health status measure [37,38]: “Would you say that in general your health is?” The 5 response categories were excellent, very good, good, fair, and poor. We dichotomized the responses: 1=fair or poor health versus 0=otherwise. The second measure, chronic condition, assessed the presence of chronic health conditions by asking participants whether they have ever been told by a health professional that they had diabetes, asthma, heart disease, cancer, or arthritis. Those reporting a diagnosis of one or more conditions were coded as 1=having at least one chronic condition versus 0=otherwise.

### Statistical Analysis

We used descriptive analyses (frequencies and percentages for categorical variables and means and standard deviations for continuous variables) to summarize and examine study data by the levels of the outcome variable (intention to use telehealth services). To take into account the ordinal nature of the outcome variable (that there is a clear ordering of the levels but with unknown absolute distances between them) and the fact that the number of the ordered levels is fewer than 5, we conducted bivariate and multivariate proportional odds regression analyses [39,40]. To identify independent factors associated with the intention to use telehealth services, we fitted proportional odds regression models with variables selected based on the bivariate analyses and controlling for potential confounders. We assessed for potential multicollinearity and 2-way interactions between the variables included in the models. Model-based adjusted odds ratios (ORs) for lower versus higher response levels for the intention to use telehealth services and their respective 95% confidence intervals were estimated. The assumption of the proportional odds model that the effects of any explanatory variables are proportional across any response levels were tested using the score test and likelihood ratio test. To select a good model, we used Akaike information criterion.

**Figure 1:**
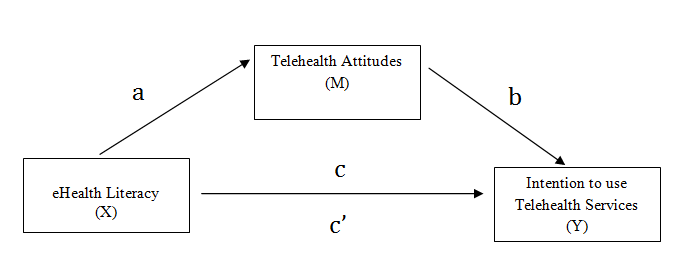
Path diagram of the hypothesized mediation model.

To test for the potential mediation effect of telehealth attitudes on the effect of eHealth literacy on the intention to use telehealth services (Figure 1), we conducted mediation analysis. A mediator is a variable M (eg, telehealth attitudes) that falls into the casual pathway between an independent variable X (eg, eHealth literacy) and an outcome variable Y (eg, intention to use telehealth services) and at least partially explains the effects of X on Y. To examine a potential mediation effect, we decomposed the total effect of eHealth literacy on the intention to use telehealth services (c path) into two causal paths, direct effect (c′ path between eHealth literacy and intention to use telehealth services not passing through telehealth attitudes) and indirect effect (path between eHealth literacy and the intention to use telehealth services passing through telehealth attitudes) [41].

The focus of the mediation analysis was to evaluate and estimate the indirect effect of eHealth literacy on the intention to use telehealth services using the product of coefficients approach, c=ab and employing the method proposed by VanderWeele et al [42] by fitting the regression models (1) and (2) below in the settings of an ordinal outcome and a continuous mediator:

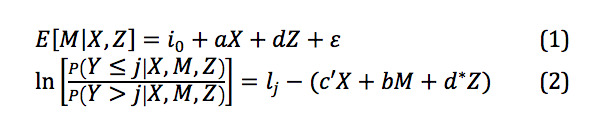



where equation (1) is a linear regression equation for the continuous mediator M on the explanatory variable X and a covariate Z with intercept *i*_0_ and slopes a and b, respectively, and a random error ε~*N*(0,σ^2^); and equation (2) is a proportional odds regression model for the log of probability of a smaller response *Y*≤*j* compared with the probability of a larger response *Y*>*j* on independent variable X with regression coefficient c′, mediator variable M with regression coefficient b, and covariate Z with regression coefficient d*, *l_j_* are the intercepts, and j=1,2,3,4 is the number of the ordered categories in the outcome variable Y.

Under the assumptions that the reference level in the outcome variable is common, the model is correctly specified, and the mediator follows a normal distribution with a constant conditional variance, the natural indirect effect (NIE) and natural direct effect (NDE) for an exposure X on the outcome Y comparing any two X=x and X=x* for a proportional odds model are given by:

NIE ≈ exp {ab(x–x*)} (**3**)

NDE ≈ exp {c′(x–x*)} (**4**)

The standard errors of the log of the aforementioned effects were estimated using the Delta method [42,43].

Proportion of mediated effect in the total effect was computed as:

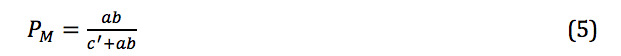



Multivariate regression and mediation analyses were conducted using complete case analysis under the missing at random (MAR) assumptions. The 294 participants used to build the model did not differ from the original sample in terms of sociodemographic characteristics: age, gender, education level, marital status, employment status, language, income, and health insurance status.

All statistical analyses were generated using SAS software version 9.4 (SAS Institute Inc). All statistical tests were 2-sided and performed at .05 significance level.

## Results

### Sample Characteristics

Table 1 reports sample characteristics by the levels of the outcome variable (intention to use telehealth services). Most of the participants were female (261/322, 81.1%), Hispanic (310/319, 97.2%), and of low socioeconomic status (219/298, 73.5% reported annual household incomes below $20,000). Only 59.3% (191/322) were high school graduates and 41.6% (131/315) were employed. Around a third (117/320, 36.6%) were born in the United States; a similar percentage (126/322, 39.1%) chose to complete the survey in English. Over half of participants (181/322, 56.2%) were married. The average age was 43 (SD 14.1) years. Not surprisingly, given the nature of the event where free health care services are the main attraction, only a fifth (65/320, 20.3%) were insured. A large proportion (136/322, 42.2%) self-rated their health as fair or poor, considerably higher than the corresponding numbers of 30% and 21% at the county and state levels, respectively [44]. Over a third (124/321, 38.6%) indicated having at least one chronic health condition.

Regarding familiarity with technology, only 59.9% (193/322) indicated having gone online to access the internet or send/receive emails. Almost 40% (118/306, 38.6%) self-rated their internet skills as not skilled at all or not very skilled. As a result, a small proportion of participants exhibited telehealth readiness: only 23.9% (77/322) reported having used technology to communicate with their health care provider or keep track of their personal health information. While only 9.4% (30/319) had heard of telehealth, once provided with a telehealth definition, 4 in 5 respondents were either very likely (137/322, 42.5%) or somewhat likely (117/322, 36.3%) to use telehealth services, if offered by their health care provider. The remainder were either not very likely (25/322, 7.8%) or indicated that they would not use these services (43/322, 13.4%). The average eHEALS score was 28 (SD 7.1, range 8 to 40) and the average telehealth attitudes score was 30 (SD 5.9, range 13 to 45).

Bivariate analyses (Table 1), using proportional odds regressions to predict lower versus higher intentions to use telehealth services, revealed that only country of birth (*P*=.01), language in which survey was administered (*P*=.01), eHEALS score (*P*=.03), and telehealth attitudes (*P*<.001) were significantly associated with the intention to use telehealth services.

**Figure 2:**
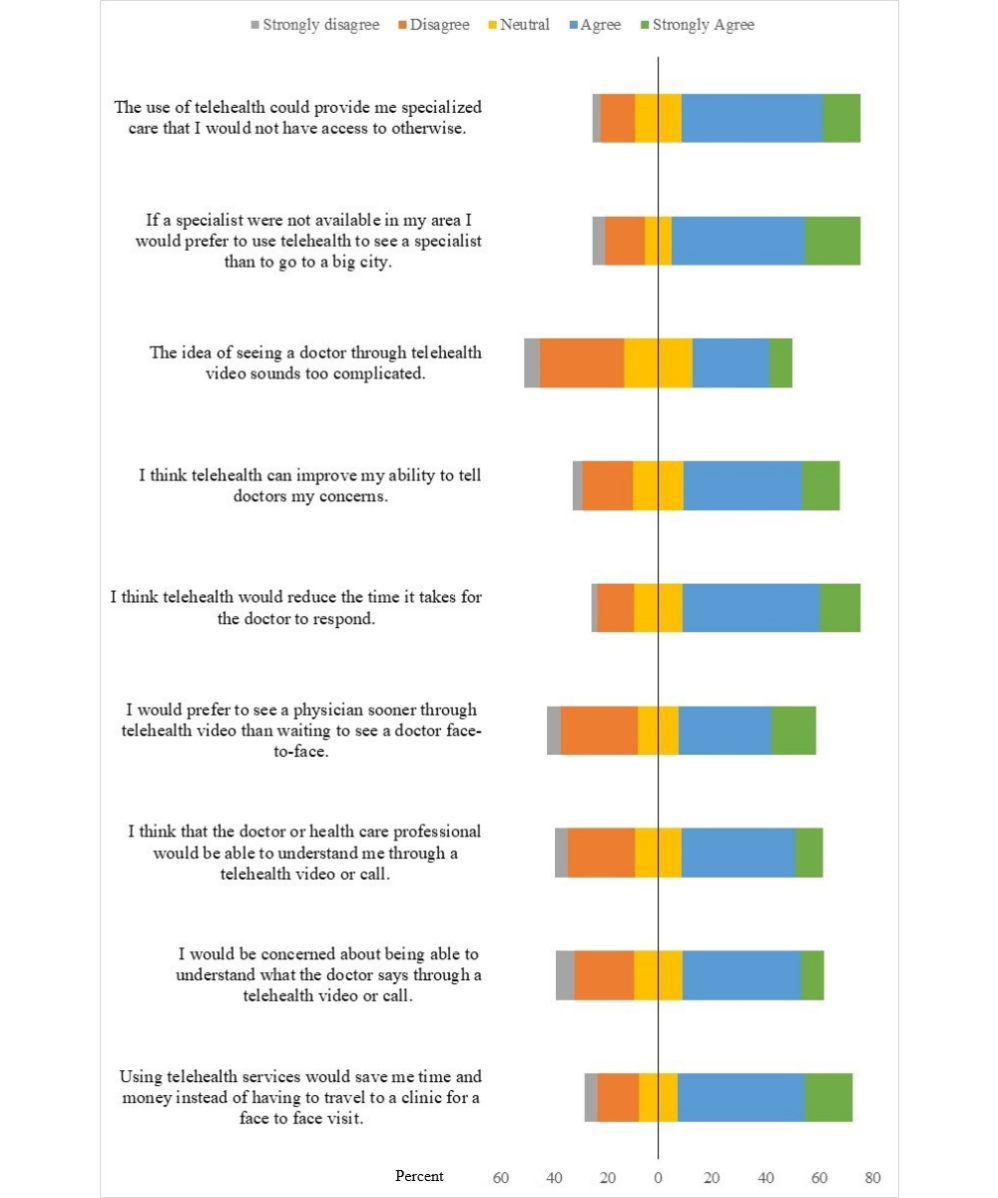
Participant responses to attitudinal statements related to telehealth use.

Overall, respondents reported positive attitudes toward the use of telehealth. The majority indicated agreement with the idea that telehealth can save time and money and provide access to specialized care (Figure 2). An area of concern for more than half of respondents (169/321, 52.6%) was related to their ability to understand the physician through a telehealth video or call.

The significant variables based on bivariate analysis, with the exception of language, were then included in a multivariate proportional odds regression model. Given the high level of collinearity between language and country of birth (*r*=.732), we opted to include only the country of birth for its more objective level of measurement relative to the language variable. The language variable was based on the language in which participants chose to complete the survey (English or Spanish); while indicative of language preference, it does not necessarily measure the level of English language proficiency or correspond to language measures in other studies or national datasets. Country of birth, on the other hand, is a standard measure and allows for comparability across studies [45]. To ensure that the language variable did not impact the results differently, we repeated the analysis with language instead of country of birth and the results were similar.

**Table 1:** Sample characteristics by the levels of the intention to use telehealth services if offered by a health care provider.

Variables			Total sample (n=322)	Very likely (n=137)	Somewhat likely (n=117)	Not very likely (n=25)	Would not use (n=43)	*P* value^a^
**Categorical, n (%)**								
	Female		261 (81)	111 (81)	91 (78)	21 (84)	38 (88)	.54
	Hispanic		310 (97)	133 (98)	112 (97)	24 (100)	41 (95)	.58
	Income^b^ <$20K		219 (74)	100 (79)	71 (65)	18 (72)	30 (81)	.33
	High school graduate		191 (59)	78 (57)	71 (61)	19 (76)	23 (54)	.56
	Employed		131 (42)	53 (40)	56 (48)	12 (50)	10 (24)	.70
	US-born		117 (37)	39 (29)	49 (42)	11 (46)	18 (43)	.01
	English survey		126 (39)	40 (29)	51 (44)	14 (56)	21 (48)	.01
	Married		181 (56)	77 (56)	66 (56)	11 (44)	27 (63)	.92
	Insured		65 (20)	29 (21)	22 (19)	4 (16)	10 (23)	.84
	**Health status**							
		Fair/poor	136 (42)	55 (40)	48 (41)	9 (36)	24 (56)	.26
		Chronic condition	124 (39)	57 (42)	42 (36)	9 (36)	16 (37)	.37
	**Internet skills**							
		Gone online^c^	193 (60)	76 (56)	77 (66)	19 (76)	21 (49)	.45
		Not/not very skilled	118 (39)	56 (43)	35 (31)	9 (38)	18 (46)	.53
	**Telehealth**							
		Telehealth ready	77 (24)	33 (24)	30 (26)	6 (24)	8 (19)	.69
		Heard of telehealth	30 (9)	11 (8)	14 (12)	4 (16)	1 (2)	.92
**Continuous, mean (SD)**								
	Age in years		43 (14)	44 (14)	41 (14)	41 (12)	46 (17)	.62
	eHEALS^d^		28 (7)	28 (7)	28 (7)	29(6)	25 (9)	.03
	Telehealth attitudes		30 (6)	34 (5)	30 (4)	28 (5)	23 (6)	<.001

^a^Wald chi-square test.

^b^Annual household income.

^c^Gone online to access the internet or to send and receive emails.

^d^eHEALS: eHealth Literacy Scale.

As shown in Table 2, eHEALS was no longer significantly associated with the intention to use telehealth (*P*=.68). The telehealth attitudes variable maintained significance, indicating that a 1-point increase in telehealth attitudes reduced the odds of lower versus higher response in the intention to use telehealth services by 23% (OR 0.77, 95% CI 0.73-0.81). In addition, US-born participants, compared with foreign-born participants, had 2.20 (95% CI 1.35-3.58) times higher odds of lower versus higher response in the intention to use telehealth services.

**Table 2:** Multivariate proportional odds regression model of factors associated with lower versus higher response to the intention to use telehealth services (n=294).

Variable	Coefficient estimate (SE)	OR^a^ (95% CI)	*P* value^b^
eHEALS^c^	0.01 (0.02)	1.01 (0.97-1.04)	.68
Telehealth attitudes	–0.28 (0.03)	0.77 (0.73-0.81)	<.001
US-born	0.79 (0.25)	2.20 (1.35-3.58)	.002

^a^OR: odds ratio.

^b^Wald chi-square test.

^c^eHEALS: eHealth Literacy Scale.

### Mediation Analysis

Bivariate analysis (Table 1) revealed that eHealth literacy, as measured by eHEALS, was significantly associated with the intention to use telehealth services (*P*=.03). After controlling for the effect of country of birth, eHEALS remained significantly associated with the outcome (*P*=.02). The assumptions of the linear regression model (telehealth attitudes regressed on eHEALS and country of birth) for normal (Shapiro-Wilk test *P* value=.07) and homoscedastic errors (White test *P* value=.16) were satisfied. Linear regression analysis showed that eHEALS was significantly associated with the hypothesized mediator, telehealth attitudes (*P*=.002), controlling for the effect of country of birth (Table 3). Based on the proportional odds regression model, eHEALS was no longer associated (*P*=.68) with the intention of telehealth use after adjusting for telehealth attitudes and country of birth (Table 3). This indicated that telehealth attitudes fully mediated the association between eHEALS and intention to use telehealth services. Using the Delta method, the estimated NIE of eHEALS on the intention to use telehealth was significant (OR 0.95, 95% CI 0.93-0.98; *P*<.001). For a 1-point increase in eHEALS, the odds of lower use of telehealth services decreased by a factor of 0.95 (5%) via the increase in the score of telehealth attitudes, controlling for the effect of country of birth. The estimated proportion of mediated effect of eHEALS in the total effect was 117.87% (Table 3). The fact that the direct effect *c*′ was opposite in sign to the indirect *ab* is known as inconsistent mediation [46] because the mediator acts like a suppressor variable. For the same reason, the estimated proportion of the mediated effect was greater than 1 [46].

**Table 3:** Adjusted estimated effects based on mediation analysis conducted with linear and proportional odds regression models (n=294).^a^

Variable	Coefficient estimate (SE)	OR^b^ (95% CI)	*P* value
eHealth literacy (*a* coefficient)	0.18 (0.05)	N/A^c^	.002
Telehealth attitudes (*b* coefficient)	–0.28 (0.03)	0.77 (0.73-0.81)	<.001
eHealth literacy (direct effect, *c′* coefficient)	0.01 (0.02)	1.01 (0.97-1.04)	.68
eHealth literacy (indirect effect, *ab* coefficients)	–0.05 (0.01)	0.95 (0.93-0.98)	<.001

^a^All effects are adjusted for country of birth.

^b^OR: odds ratio.

^c^Coefficient was estimated using linear regression.

## Discussion

### Principal Findings

This study examined the attitudes of vulnerable minority groups toward telehealth and assessed the factors that shape their intention to use telehealth services. Findings revealed that marginalized Hispanic communities along the Texas-Mexico border have had limited exposure to telehealth. Despite that, participants exhibited generally positive attitudes toward telehealth which, in turn, were associated with a higher likelihood of using telehealth services if offered by one’s health care provider. Additionally, these positive attitudes mediated the relationship between eHealth literacy and the intention to use telehealth, highlighting the important role that eHealth literacy plays in shaping attitudes and, ultimately, telehealth acceptance.

The positive association between attitudes toward telehealth and intention to use telehealth services is in line with the basic concept underlying the different technology acceptance frameworks [7,47,48], where reactions to using a certain information technology (attitudes, perceived usefulness, perceived ease of use) impact the behavioral intention to use that technology. Studies specific to HIT also support that pathway [10,25,26].

While health information technology acceptance and adoption models have evolved over time to integrate end-user characteristics, these models have rarely considered eHealth literacy. Kim and Park [10] include the technological/computer literacy domain of eHealth literacy [49,50], which they term as HIT self-efficacy. In their model, HIT self-efficacy is conceptualized to affect perceived usefulness and ease of use, which in turn shape attitudes and the behavioral intention to use HIT. Our findings are in line with that causal pathway, although eHealth literacy is a much broader concept that encompasses many other skills beyond technological literacy and includes functional, communicative, critical, and transactional eHealth literacy skills [49]. This highlights the need to integrate eHealth literacy, with its multidimensional characteristics, in HIT conceptual frameworks. This is especially relevant as the evidence is mounting on eHealth literacy’s role in extending the digital divide to health care [51,52] and, just as importantly, in facilitating the adoption of various eHealth apps. For example, our finding of the significance of eHealth literacy to telehealth use intention mirrors other findings where eHealth literacy has been found to have a significant association with the use of or the intention to use other consumer eHealth platforms such as patient portals [13,14] and mHealth [15,16].

### Limitations

There are several limitations to this study. First, our outcome variable, the behavioral intention to use telehealth, was assessed by the question, “How likely are you to use telehealth services if they were offered by your provider?” This may be interpreted to assume that a participant has a health care provider, a questionable premise given the high rate of uninsurance in our sample. However, it is worth noting that despite being uninsured, participants interact with health care providers in a variety of traditional and nontraditional settings. Some have providers through local safety net clinics; data collected at the same event in 2015 from participants with an almost identical sociodemographic profile showed that 54% of the uninsured had received health care services at the local county health clinic [53]. Area residents also seek health care services across the border in Mexico; a population-based survey of 1405 Texas border county residents revealed that respondents, especially the uninsured, regularly sought health care services in Mexico, with 38% of respondents reporting a doctor’s visit within the past 12 months [54]. Furthermore, many uninsured community members interact with health care providers for their children’s health care services; while 43% of Hidalgo County’s population between the ages of 18 and 64 years did not have health care coverage in 2018, the corresponding rate for those under age 19 years was 13.6% [32], reflecting the higher insurance rates among children through the Children’s Health Insurance Program (CHIP) or children’s Medicaid.

Second, similar to many health information technology acceptance and adoption studies, data were collected at a single time point, with no experimental manipulation or random assignment, resulting in the inability to establish causality.

Third, to our knowledge, there are no previous studies that evaluate the causal relationship between eHealth literacy, telehealth attitudes, and intention to use telehealth services. Therefore, our mediation analysis was not based on a theoretically defined causal chain of variables. However, some models depicting the causal pathway between end-user characteristics and the intention to use HIT include variables that can serve as proxies for eHealth literacy such as HIT self-efficacy [10]; the depicted causal pathways in these models support our findings. It is worth noting that these studies also use cross-sectional data.

Fourth, our introduction of telehealth to participants did not take into account the cost of telehealth services, a highly relevant factor for poor, uninsured communities. Another limitation is that most participants had not heard of telehealth. Thus, attitudes and the intention to use telehealth reflected a hypothetical scenario to most participants. Nevertheless, such positive attitudes point to a window of opportunity that can be reinforced by well-designed virtual platforms that take into account the eHealth literacy skills of the target population.

Finally, the sociodemographic homogeneity of the sample did not allow for capturing the impact of ethnicity, educational attainment, socioeconomic status, and other sociodemographic factors on the intention to use telehealth; such factors have been shown to exhibit varying levels of influence on the use of eHealth [55]. However, given our focus on vulnerable minority groups, this homogeneity allows us to better control for such effects. One exception is that the overrepresentation of females in our sample, a reflection of event attendance, may limit the generalizability of our findings to males. While the higher representation of women relative to men at the event may be an indication of working schedules and women assuming responsibilities for child immunizations and physicals, it may also be reflective of the well-documented gender differences in accessing health care and adhering to preventive care guidelines [56,57]. Such differences may potentially extend to the intentions to use telehealth services.

### Future Research

Multiple venues exist to expand our knowledge on telehealth use. First, it is important to strengthen the evidence on the causal pathways leading to telehealth adoption by incorporating longitudinal research designs [9]. Although our mediation analysis only showed mathematically that telehealth attitudes were a significant mediator of the effect of eHealth literacy on the intention to use telehealth services, this finding provides a strong basis for testing the causal link through future longitudinal data or experimental designs. In addition, it is important to expand the literature on the intention to use telehealth to include actual adoption and use. The recent adoption of telehealth due to the COVID-19 pandemic provides a fertile ground for research on the facilitators and barriers to telehealth use in vulnerable communities. Future research should also address the development of attitudinal measures toward telehealth that exhibit good psychometric properties. Most studies in the literature, similar to our study, use various sets of statements that reflect the particular research question and setting [28,58,59]. Furthermore, it is important to incorporate additional factors such as social influence [9] and privacy and data security issues [60] and explore their impact on telehealth acceptance and adoption. The latter is especially important since perceived privacy and security concerns have been shown to impact the intention to use telehealth services [61]. Finally, it is essential to explore telehealth’s role in extending services to the uninsured and investigate delivery and reimbursement models specifically targeting this population.

### Conclusions

To our knowledge, this study is among the first to focus primarily on a vulnerable Hispanic population and use mediation analysis to explore the role of eHealth literacy on the behavioral intention to use telehealth. Our findings contribute significantly to promoting telehealth adoption in communities where access to health care services is a major challenge and an understanding of end-user characteristics is key to successful intervention design and adoption.

Telehealth promises to address many of the access challenges facing ethnic and racial minorities, rural communities, and low-income populations. Understanding the factors that influence its acceptance and, subsequently, its adoption is an essential first step to designing culturally relevant platforms that take into account key characteristics of these communities. Raising awareness about telehealth and developing interventions that target eHealth literacy skills promise more positive attitudes and more willingness to engage in telehealth use.

## Abbreviations

CDC: Centers for Disease Control and Prevention
CoPHII: Collaborative on Population Health Innovation and Improvement
eHEALS: eHealth Literacy Scale
eHealth: electronic health
HIT: health information technologies
MAR: missing at random
mHealth: mobile health
NDE: natural direct effect
NIE: natural indirect effect
OLS: Operation Lone Star
OR: odds ratio
PSJA: Pharr-San Juan-Alamo
UTRGV: University of Texas Rio Grande Valley
